# Supplementary material for: Severe COVID-19 patients have impaired plasmacytoid dendritic cell-mediated control of SARS-CoV-2
Source: Nat Commun. 2023 Feb 8;14:694. doi: 10.1038/s41467-023-36140-9 (PMC9907212; doi:10.1038/s41467-023-36140-9)
Supplement: Supplementary file 4 — Source Data [file 41467_2023_36140_MOESM4_ESM.zip › Source data/Venet Fig 2b_pDCs_def/2022-04-28_Thu_16-52-33_GradientBoostClassifier_6/Confusion_matrix_Downsampling 8.pdf]

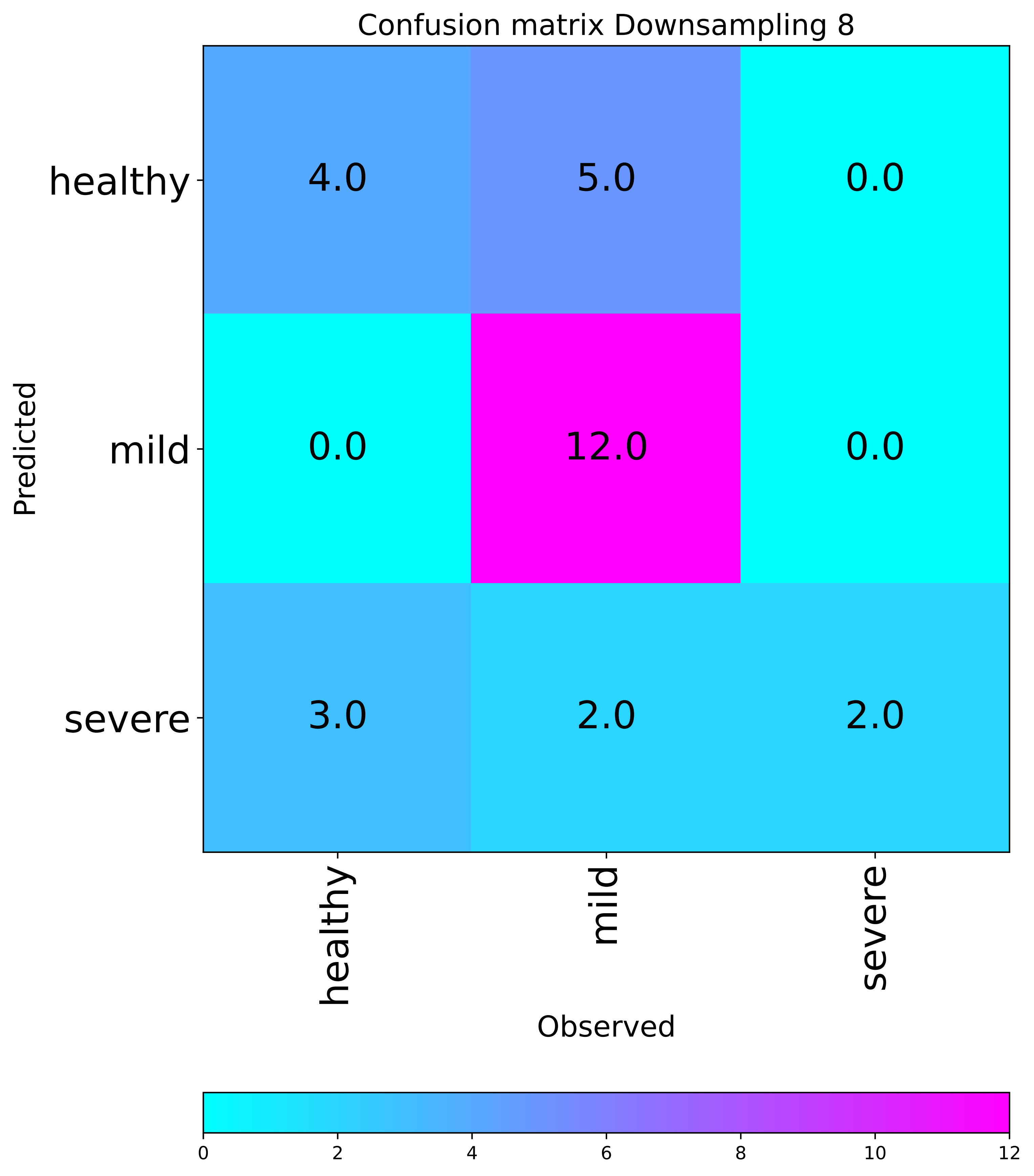

Confusion matrix Downsampling 8
Predicted
healthy
mild
severe
4.0
5.0
0.0
0.0
12.0
0.0
3.0
2.0
2.0
healthy
mild
severe
Observed
0
2
4
6
8
10
12
